# Supplementary material for: Optineurin is a gatekeeper of mitochondrial health and proteostasis in Alzheimer’s disease vulnerable neurons
Source: bioRxiv. 2026 Mar 16:2026.03.15.711617. Preprint. [Version 1] doi: 10.64898/2026.03.15.711617 (PMC13015401; doi:10.64898/2026.03.15.711617)

bioRxiv preprint doi: https://doi.org/10.64898/2026.03.15.711617; this version posted March 16, 2026. The copyright holder for this preprint (which was not certified by peer review) is the author/funder, who has granted bioRxiv a license to display the preprint in perpetuity. It is made available under aCC-BY 4.0 International license.

Supplementary Figure 1. A) Top 5 genes connected to each NetWAS 2.0. module and their enrichment score in ECII neurons (ECII) compared to neurons resilient to AD. **B)** Gene expression levels of *Optn* and *Piezo1* throughout the mouse lifespan in different brain neurons from bacTRAP data. Source: alz.princeton.edu.

Supplementary Figure 2. A) Schematic representation of the *in vitro* proteomics experiments. **B)** Western blots showing Optineurin levels at 4 and 5 days post-transduction in whole cell homogenates. **C)** Western blots showing efficient subcellular fractionation of cytoplasm and synaptic compartments. **D)** PCA plot showing the separation between subcellular compartments at 4 and 5 days post-transduction. **E)** PCA plots showing the separation between control and *Optn*-silenced conditions for each of the compartments at 4 and 5 days post transduction. * pval < 0.05, Unpaired t-test with Welch's correction.

Figure S1

A

bioRxiv preprint doi: https://doi.org/10.64898/2026.03.15.711617; this version posted March 16, 2026. The copyright holder for this preprint (which was not certified by peer review) is the author/funder, who has granted bioRxiv a license to display the preprint in perpetuity. It is made available under aCC-BY 4.0 International license.

Module 0	ECII	Module 1	ECII	Module 2	ECII	Module 3	ECII
YPEL5	0.20	PIEZO1	1.67	AASDHPPT	0.10	DEK	0.04
SERINC3	0.24	NPC2	0.12	KIF3A	0.20	TMX1	0.05
OPTN	0.96	PLIN3	-	SPAST	0.08	PRKD3	-
CYLD	0.26	ANXA5	-	C2CD5	0.34	RECQL	0.07
GOLGB1	0.12	MYL12A	-	MATR3	0.10	MOB1A	-

B

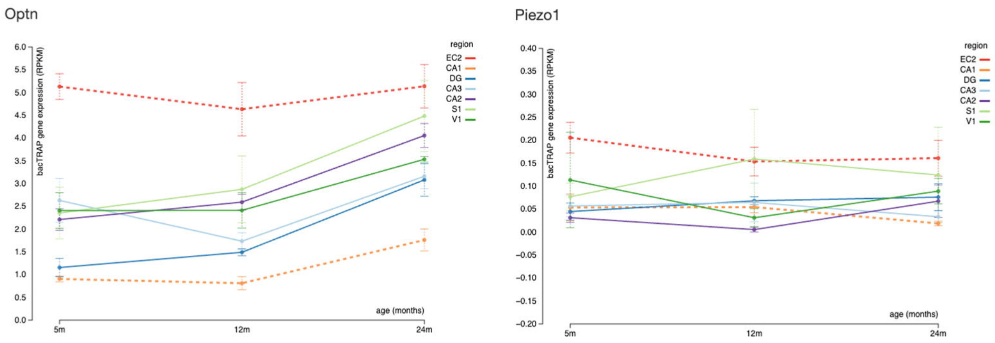

bioRxiv preprint doi: https://doi.org/10.64898/2026.03.15.711617; this version posted March 16, 2026. The copyright holder for this preprint (which was not certified by peer review) is the author/funder, who has granted bioRxiv a license to display the preprint in perpetuity. It is made available under aCC-BY 4.0 International license.

Figure S2

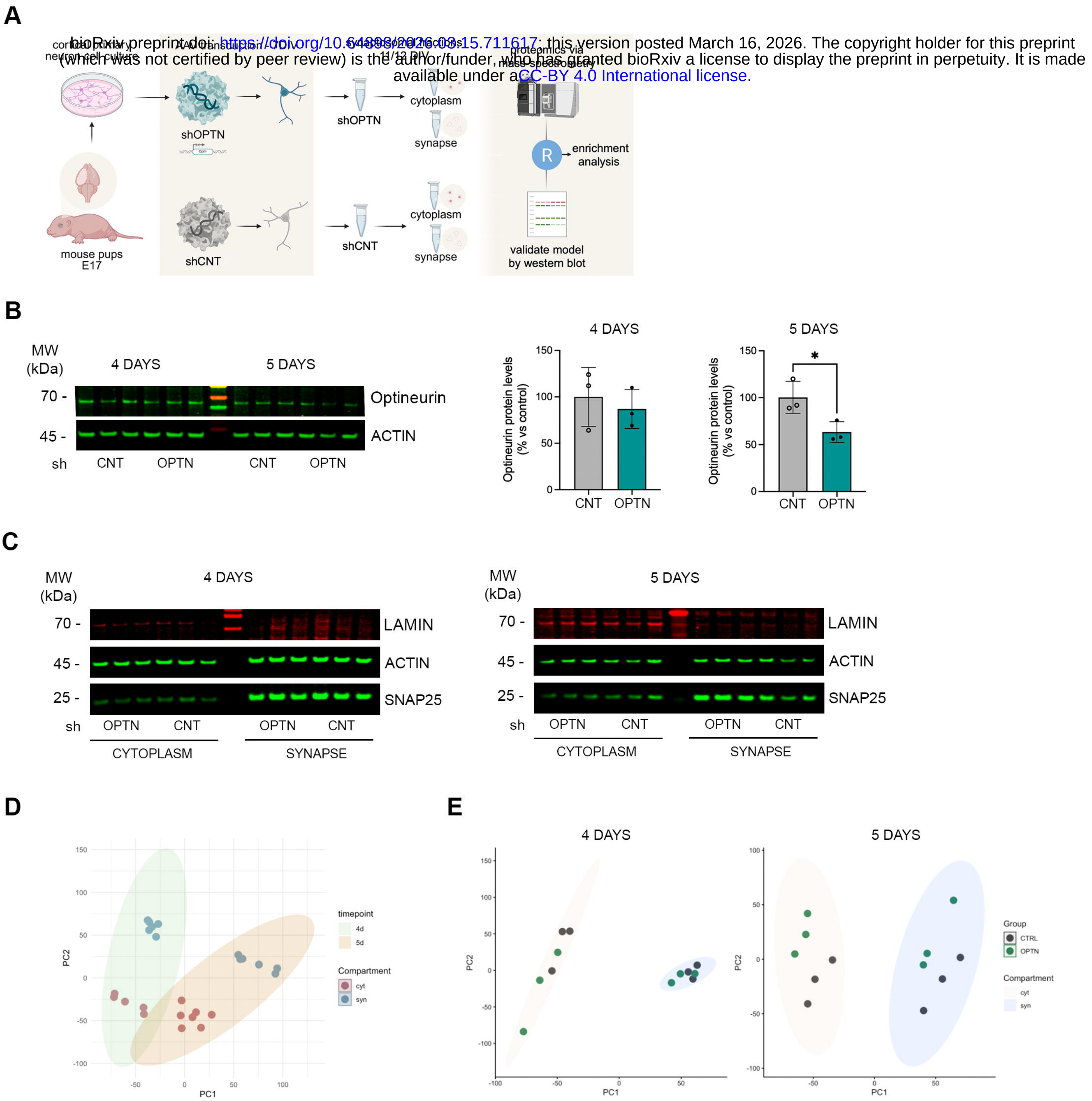

Supplement: Supplement 5 [file NIHPP2026.03.15.711617v1-supplement-5.pdf]
